# Immature monocyte derived dendritic cells gene expression profile in response to Virus-Like Particles stimulation

**DOI:** 10.1186/1479-5876-3-45

**Published:** 2005-12-29

**Authors:** Eleonora Aricò, Ena Wang, Maria Lina Tornesello, Maria Tagliamonte, George K Lewis, Francesco M Marincola, Franco M Buonaguro, Luigi Buonaguro

**Affiliations:** 1 Immunogenetics Section, Department of Transfusion Medicine, Clinical Center, National Institutes of Health, Bethesda, MD 20892-1502, USA; 2 Department of Cell Biolology and Neurosciences, Istituto Superiore di Sanita, Rome, Italy; 3 Lab. Viral Oncogenesis and Immunotherapies & AIDS Reference Center, Department of Experimental Oncology, Istituto Nazionale Tumori "Fond. G. Pascale", 80131 Napoli, Italy; 4 Institute of Human Virology, University of Maryland Biotechnology Institute; 5 Department of Microbiology and Immunology, University of Maryland School of Medicine, University of Maryland Baltimore, Baltimore, MD 21201, USA

## Abstract

We have recently developed a candidate HIV-1 vaccine model based on HIV-1 Pr55gag Virus-Like Particles (HIV-VLPs), produced in a baculovirus expression system and presenting a gp120 molecule from an Ugandan HIV-1 isolate of the clade A (HIV-VLP_A_s).

The HIV-VLP_A_s induce in Balb/c mice systemic and mucosal neutralizing Antibodies as well as cytotoxic T lymphocytes, by intra-peritoneal as well as intra-nasal administration. Moreover, we have recently shown that the baculovirus-expressed HIV-VLPs induce maturation and activation of monocyte-derived dendritic cells (MDDCs) which, in turn, produce Th1- and Th2-specific cytokines and stimulate *in vitro *a primary and secondary response in autologous CD4+ T cells.

In the present manuscript, the effects of the baculovirus-expressed HIV-VLP_A_s on the genomic transcriptional profile of MDDCs obtained from normal healthy donors have been evaluated. The HIV-VLP_A _stimulation, compared to both PBS and LPS treatment, modulate the expression of genes involved in the morphological and functional changes characterizing the MDDCs activation and maturation.

The results of gene profiling analysis here presented are highly informative on the global pattern of gene expression alteration underlying the activation of MDDCs by HIV-VLP_A_s at the early stages of the immune response and may be extremely helpful for the identification of exclusive activation markers.

## Introduction

Virus-like particles (VLPs) represent a peculiar form of subunit vaccine based on viral capsid and envelope proteins which show the ability to self-assemble into highly organized particulate structures [1,2]. VLPs closely resemble immature virus particles but are both replication and infection incompetent, lacking regulatory proteins as well as infectious genetic material. VLPs can be employed to deliver additional antigenic structures, such as whole proteins or specific individual epitopes and have been shown to generally induce more effective humoral and cellular immune response than their soluble counterparts [3].

Considering all these properties, VLPs represent a highly attractive vaccine approach and have been produced from a broad spectrum of enveloped and non-enveloped viruses, regardless of whether the particle structure is based on single or multiple capsid proteins [4].

The VLPs developed in our laboratory are based on the Human Immunodeficiency Virus type 1 Pr55gag precursor protein (HIV-VLPs) and present an entire gp120 molecule, anchored through the trans-membrane (TM) portion of the Epstein-Barr virus (EBV) gp220/350 [5]. The gp120 glycoprotein selected for these HIV-VLPs derives from an Ugandan HIV-1 isolate of the A clade [6,7], which represents the second most prevalent HIV-1 subtype worldwide (approx. 25%) and is predominant in many developing countries (HIV-VLP_A_s).

The HIV-VLP_A_s show a strong in vivo immunogenicity in Balb/c mice, even in absence of adjuvants, and HIV-1-specific T cell response (CD4+ and CD8+) as well as cross-clade neutralizing antibodies have been detected in immunized animals [8]. Moreover, the intra-peritoneal and the intra-nasal administrations of HIV-VLP_A_s induce in mice an antibody response at systemic as well as local (vaginal and intestinal) level [9].

Most of the VLPs developed have been shown to be highly effective at stimulating CD4 proliferative responses and cytotoxic T lymphocyte (CTL) responses in addition to B-cell-mediated humoral immunity [4]. These properties suggest the ability to promote the activation of antigen-presenting cells (APCs) and a cross-presentation of peptides in association to both MHC class I and -II molecules [10,11].

We have recently shown that baculovirus-expressed HIV-VLP_A_s are able to induce maturation of DCs, resulting in expression of surface maturation markers as well as increased production of Th1 polarizing cytokines, and this effect is partially mediated by the intra-cellular TLRs 3 and 8. The HIV-VLP-activated DCs induce a primary and secondary response in autologous CD4+ T cells, in an *in vitro *immunization assay. Finally, the uptake of HIV-VLPs by DCs appears to be mainly mediated by an endocytosis-mediated pathway (Buonaguro L, *et al*., submitted).

Dendritic cells (DCs) are professional antigen-presenting cells (APC) able to initiate immune responses [12,13]. Immature DCs are located in peripheral tissues to continuously monitor the environment through the uptake of particulate and soluble products. Antigen-loaded DCs acquire a mature phenotype, associated with reduced endocytic and phagocytic capacities, and enhanced production of inflammatory cytokines and chemokines [14-17]. The mature DCs, then, migrate toward the lymphoid organs where they interact with, and activate, naïve T cells [18].

The analysis of the transcription profile, defined as transcriptome, is highly informative of the molecular basis underlying the morphological, phenotypical and functional changes of specific immune cell populations induced by specific stimuli. In particular, gene-expression profiles of human Th1 and Th2 cells have allowed the identification of genetic patterns involved in the differential T helper cell polarization [19]. Similarly, selected genes differentially regulated during the transition from a B cell to plasma cell have been identified, which are involved in the Ab secretion, homeostasis, migration, and differentiation [20].

More recently, the expression pattern of specific sets of genes upon DC differentiation and maturation has been reported, showing a great plasticity of the DC transcriptional programs, activated in response to CD40L, LPS and cocktail of inflammatory cytokines and prostaglandin (PG) E(2) (CyC) [21,22]. Furthermore, a time-specific kinetic of response has been observed in MDDC activated with pathogen components, showing a rapid upregulation of genes associated with the innate arm of the immune response, followed by induction of adaptive immune response genes [23-25].

In this study, we have analyzed the changes in the gene expression of immature human monocyte-derived DC (MDDC) activated with the baculovirus-expressed HIV-VLPs developed in our laboratory. The transcriptional pattern has been evaluated after 4 and 8 hours of induction with HIV-VLPs and the results show the sustained increased expression of specific genes involved in the morphological and functional changes characterizing the MDDCs activation and maturation.

## Materials and methods

### Cell culture medium

DC culture medium consisted of RPMI 1640 medium (Life Technologies, Carlsbad, Calif.) supplemented with 2 mM L-glutamine (Sigma), 1% nonessential amino acids (Life Technologies), 1% sodium pyruvate (Life Technologies), 50 μM 2-mercaptoethanol (Sigma), 50 μg of gentamicin (Life Technologies) per ml, and 10% fetal calf serum (Life Technologies).

### DC preparation and treatment

All human specimens were obtained under informed consent, as approved by the University of Maryland Baltimore Institutional Review Board. Monocyte-derived DCs were generated as described previously (17), with minor modifications. Briefly, human peripheral blood mononuclear cells were isolated, from three independent normal healthy donors, by Ficoll-Hypaque density gradient centrifugation and were enriched for CD14^+ ^monocytes by negative selection with a cocktail of monoclonal antibodies from StemCell Technologies (Vancouver, British Columbia, Canada), according to the instructions of the manufacturer. Typically, greater than 80% of the cells were CD14^+ ^after enrichment (data not shown). The isolated monocytes were allowed to adhere to plastic by plating 10^6 ^cells per/ml in RPMI 1640 medium for 2 h. Adherent monocytes were washed with RPMI 1640 medium and were then cultured for 6 days at 10^6 ^cells per/ml in DC culture medium supplemented with 50 ng of recombinant GM-CSF (rGM-CSF, R&D Systems, Minneapolis, Minn.) per ml and 1,000 U of recombinant IL-4 (rIL-4; R&D Systems, Minneapolis, Minn.) per ml.

After 6 days in culture, MDDCs were pulsed with either 5 μg/ml of HIV-VLPs or 1 μg/ml of LPS for 4 and 8 hours, for gene microarray analysis, and for 16 hours for maturation and activation phenotype analysis.

### Analysis of DC phenotype

DCs were incubated for 30 min at 4°C with murine monoclonal antibodies specific for CD80, CD83, CD86, and HLA-DR (BD Pharmingen, San Diego, CA), washed and then fixed with 2% paraformaldehyde for analysis with a FACScalibur flow cytometer (BD Pharmingen). Data analysis was carried out with FlowJo software (Tree Star Inc., San Carlos, CA). The fraction of MDDCs that responded by upregulation of activation markers on the cell surface was calculated by overlaying the histograms of treated and untreated MDDCs and Overton subtraction of the curves.

### RNA preparation and microarray hybridization

DCs were harvested, washed twice in PBS and lysed in 350 ul RLT buffer with fresh addition of 2-Mercaptoethanol per each well of the 6-well plate. Total RNA was isolated using RNeasy minikits (Qiagen), according to the manufacturer's protocol, and RNA quality and quantity was estimated by Agilent Bioanlayzer (Agilent Technologies, Palo Alto, CA) and NonoDrop. Amplified antisense RNA (aRNA) was obtained from total RNA (0.5–3 μg) via two round of in vitro transcription, according the protocol previously described by us [26]. 6 ug of amplified test samples aRNA were labeled with Cy5 (Amersham) while the same amount of reference sample (pooled normal donor PBMCs) was labeled with Cy3. Test-reference sample pairs were mixed and co-hybridized to 17K cDNA microarrays [27].

### Microarrays and statistical analyses

Hybridized arrays were scanned at 10-μm resolution on a GenePix 4000 scanner (Axon Instruments) at variable PMT voltage to obtain maximal signal intensities with less than 1% probe saturation. Resulting jpeg and data files were deposited at microarray data base (mAdb)  and retrieved after median centered, filtering of intensity (>300) and spot elimination (bad and no signal). Data were further analyzed using Cluster and TreeView software [27] and Partek Pro software (Partek). Subsequent low-stringency filtering (80% gene presence across all experiments and at least one experiment with ratio fold change >3), 3,119 genes were selected for further analysis. Hierarchical cluster analysis was conducted on these genes according to Eisen *et al*. [28]; differential expressed genes were visualized by Treeview and displayed according to the central method [29].

## Results

### Baculovirus-HIV-VLP induces a maturation phenotype of DCs

**Figure 1:**
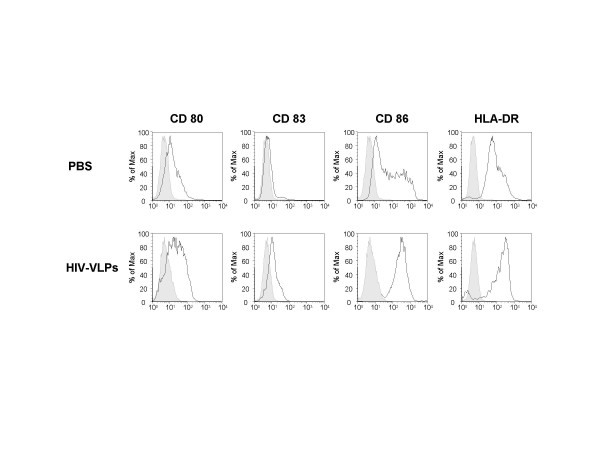
**Maturation of DCs by baculovirus-expressed HIV-VLPs**. Immature MDDCs were incubated in the presence of the indicated stimulus for 16 hours. The expression of CD80, CD83, CD86 and HLA-DR was analyzed on fixed cells by FACScalibur flow cytometer and data analysis was carried out with FlowJo software. The results of a representative experiment are shown; the shadowed curve represents the untreated cells.

Immature MDDCs were obtained from three independent donors and, after 6 days of culture in IL-4- and GM-CSF-enrichment medium, were incubated with 5 μg/ml of baculovirus-expressed HIV-VLPs or 1μg/ml of LPS. In parallel, control MDDCs were loaded with PBS. After a 16 hr-induction, the expression of surface maturation/activation markers, such as CD80, CD83, CD86 and HLA-DR, was examined. The expression of all the four markers was upregulated by treatment with HIV-VLPs, compared to PBS (Fig. 1). The level of cytokines involved in the Th1/Th2 polarization was assessed in the supernatant of MDDCs loaded with HIV-VLPs or LPS. In particular, the TNF-α, IL-12 p70, IL-10 were produced at higher levels in HIV-VLP-loaded MDDCs compared to LPS-loaded MDDCs (data not shown).

These results indicate that baculovirus HIV-VLPs induce a specific MDDC maturation pathway, distinct from the one induced by the LPS.

### Pattern of MDDCs response to HIV-VLPs

Gene expression profiles were generated from treated and control MDDC harvested 4 and 8 hrs after stimulation. These time points were selected to evaluate a possible biphasic response, as described for LPS-induced DCs and mononuclear phagocytes (MPs) [23,25]. Amplified antisense RNA (aRNA) was obtained from total RNA extracts [26] and hybridized to a custom-made 17,000 (17K)-clone cDNA microarray chip enriched with genes relevant to immune function. Stringent filtering were further applied to eliminate genes with missing value in >20% of all the experiments and >3 fold change in at least one experiment. The remaining 3,119 genes were thus used for statistic analysis.

**Figure 2:**
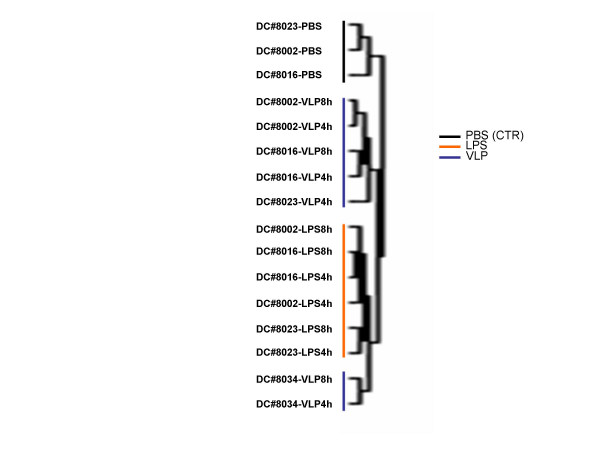
**Unsupervised hierarchical clustering of all filtered data**. The clusterogram represents 3,119 genes obtained by Eisen hierarchical clustering of the complete 17K dataset filtered for genes that are expressed in a minimum of 80% of the samples 4 and 8 h after HIV-VLPs or LPS stimulation. The PBS treatments was evaluated after 8 h of stimulation. The clustering is defined by the dendrogram and each treatment/time point is represented by a single branch.

Unsupervised cluster analysis obtained on MDDCs stimulated for 4 and 8 hr with HIV-VLPs or LPS shows the segregation of the untreated (PBS) and the treated (HIV-VLPs or LPS) MDDCs in two independent clusters. However, within the treated MDDCs cluster, the samples subclustered according to the treatment (HIV-VLPs vs LPS), with the exception of a single HIV-VLP-treated sample which formed an independent cluster closer to the LPS-treated sample, indicating the induction of two distinct transcription machinery. Furthermore, while the HIV-VLP-treated MDDCs clustered according to the donor, the LPS-treated MDDCs clustered preferentially according to the treatment duration (4 or 8 hr) of stimulation (Fig. 2). This would suggest an individual donor susceptibility to HIV-VLP-treatment and a possible delayed transcription or secondary response to LPS treatment.

### Gene expression changes induced in MDDCs by HIV-VLP treatment

The differential gene expression in HIV-VLP-treated MDDCs, compared to either LPS- or PBS-treated MDDCs, was considered statistically significant only when supported by a p < 0.005.

**Figure 3:**
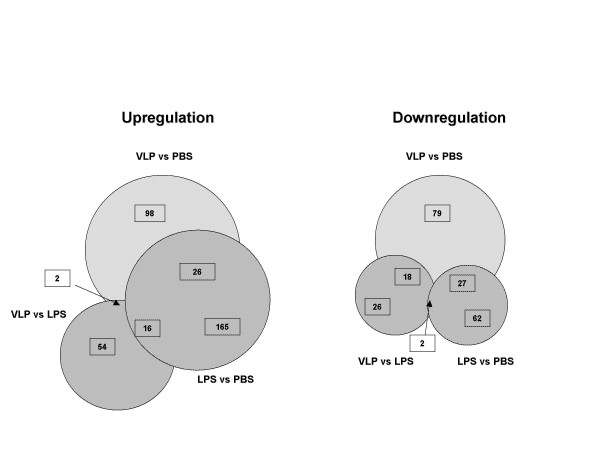
**Pattern of gene expression in human monocyte-derived DCs**. Regulated genes by HIV-VLPs or LPS treatment in MDDCs, showing at least a 2-fold modulation (up or downregulation), have been evaluated. Each circle represents the whole set of genes identified in the indicated comparisons, based on the 2-fold modulation parameter. Numbers in overlapping regions represent common regulated genes. Numbers in non-overlapping regions represent unique regulated genes. Circles are drawn in arbitrary scale.

Treatment-induced changes in gene profiling were analyzed using student t test, based on simple two-treatment comparison or one versus a combined two-treatment comparison. Considering only genes showing at least a 2-fold modulation (increase or reduction) in the transcriptional levels, it has been possible to identify unique and common genes in the profile induced by the different treatments. In particular, the HIV-VLP treatment, induced the upregulation of 140 and 72 genes as well as the downregulation of 108 and 46 genes, compared to PBS and LPS respectively. A sub-set of upregulated and downregulated genes are specific to the HIV-VLP treatment (Fig. 3), indicating that, besides transcriptional patterns shared with the LPS treatment, HIV-VLPs induce a specific reprogramming of the MDDCs transcriptional profile.

**Figure 4:**
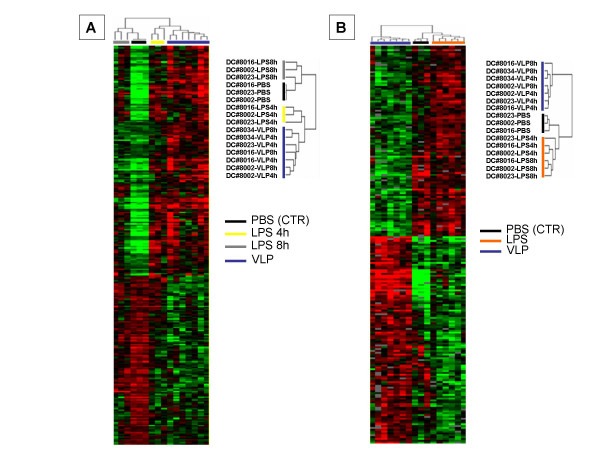
**Supervised hierarchical clustering of genes differentially expressed in HIV-VLP-treated MDDCs**. The clusterograms represent an Eisen hierarchical clustering of the 281 genes differentially expressed (p < 0.005) in HIV-VLPs-treated MDDCs, compared to PBS treatment **(A)**, and of 217 genes differentially expressed (p < 0.005) in HIV-VLPs-treated MDDCs, compared to LPS treatment **(B)**. The clustering is defined by the dendrogram on the top and on the side of the clusterogram.

Visualization of the gene expression among different treatments revealed a transcriptional profile pattern in HIV-VLP-induced MDDCs distinct from either PBS or LPS (4 hr and 8 hr) induction (Fig. 4A). Furthermore, clustering analysis performed based on 217 genes selectively expressed (p < 0.005) in HIV-VLP induced MDDCs, compared to LPS treatment, confirmed a distinct transcriptional profiles signature according to the treatment, regardless of the length of stimulation (Fig. 4B).

### Pathways modulation in MDDCs in response to HIV-VLPs

**Figure 5:**
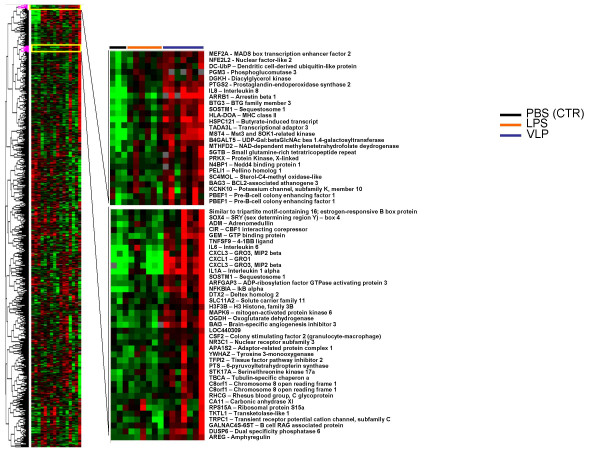
**Supervised hierarchical clustering of genes upregulated by HIV-VLPs in MDDCs**. 3,119 genes included in this analysis were filtered upon the criteria of showing less than 20% missing data and a minimum of 3-fold modulation in expression. The expanded section shows nodes including genes upregulated by HIV-VLPs. Individual genes are indicated on the right.

Gene expression changes in response to stimuli are generally pathways directed. In order to analyse pathways modulated in HIV-VLPs induced MDDCs, we dissected genes according to cluster nodes derived on the basis of expression similarity. Supervised cluster analysis based on the 3,119 genes were further analyzed according to pattern recognition. Genes uniquely upregulated in MDDCs by HIV-VLPs only or by HIV-VLPs and LPS (either at 4 h or 8 h post-induction) are indicated in Fig. 5A and 5B respectively. Similarly, nodes were identified including genes down-regulated by HIV-VLPs only or by HIV-VLPs and LPS at 4 h post-induction (Fig. 5C top and bottom, respectively). Most of the genes present in these nodes are strictly associated to the functional and morphological changes characterizing the MDDCs maturation and activation process.

In this regard, the MDDCs transcriptional profile was analyzed on the basis of the cellular pathways modulated by the HIV-VLP-treatment, compared to either PBS or LPS treatment. In respect to the main focus of this study, only the pathways involved in the MDDCs-activation by HIV-VLPs have been evaluated in more detail. These can be divided into main super-families concerning the cell replication/death, the transcriptional control, the phagocytosis and pathogen recognition, the immunological function of dendritic cells, the migration and the control of the inflammatory responses (Table I).

**Table 1:** Pathways involved in the HIV-VLPs-activated MDDCs. The pathways are derived from the BioCarta through the Cancer Genome Anatomy Project at . Genes with at least a 2-fold modulation (up or downregulation) have been taken into consideration. Upregulated genes in **bold **and underline; Downregulated genes in plain text.

**Pathways**	**HIV-VLP vs PBS**	**HIV-VLP vs LPS**	**Pathways**	**HIV-VLP vs PBS**	**HIV-VLP vs LPS**	**Pathways**	**HIV-VLP vs PBS**	**HIV-VLP vs LPS**
**Cell cycle, death**			**Transcription**			**Immunology/Signaling**		
Cadmium induces DNA synthesis and proliferation in macrophages	**NFKB1** MAPK1	TNF	Acetylation and Deacetylation of RelA in The Nucleus	**NFKB1** FADD	**NFKBIA** **TNF** FADD	AKT Signaling Pathway	**FOXO3A** **NFKB1** CASP9 YWHAH	
Caspase Cascade in Apoptosis	**BIRC3** **CASP7** CASP2 CASP9 PARP1		Activation of PKC through G protein coupled receptor	**GNAQ** **NFKB1**		B Lymphocyte Cell Surface Molecules	**CD40** **FCGR2A** **ICAM1** **LTBR** ITGAL PTPRC	**NFKBIA**
Free Radical Induced Apoptosis	**GSR** **IL8** **NFKB1**		Chaperones modulate interferon Signaling Pathway	**DNAJA3** **IFNG** **NFKB1**	**IFNG** **LIN7A** **NFKBIA** **RB1** **TNF**	CD40L Signaling Pathway	**CD40** **DUSP1** **TNFAIP3** **TRAF6** IKBKB	**DUSP1** **MAP3K1** **NFKBIA** **TNFAIP3**
Induction of apoptosis through DR3 and DR4/5 Death Receptors	**BID** **CASP10** **CASP7** **TRAF2** CASP9 FADD		Double Stranded RNA Induced Gene Expression	**NFKB1**		CTL mediated immune response against target cells	**ICAM1** **B2M** **LTBR** ITGAL	
Neuropeptides VIP and PACAP inhibit the apoptosis of activated T cells	**GNAQ** **NFKB1** **PRKAR1B** CAMK1		Human Cytomegalovirus and Map Kinase Pathways	**MAP2K3** **NFKB1** MAP2K6 MAPK1		fMLP induced chemokine gene expression in HMC-1 cells	**FPR1** **MAP2K2** **NFKB1** CAMK1 MAP2K6 MAPK1	
Regulation of BAD phosphorylation	**BCL2L1** **IGF1R** **PRKAR1B** MAPK1 RPS6KA1 YWHAH		NFkB activation by Nontypeable Hemophilus influenzae	**DUSP1** **EP300** **MAP2K3** **NFKB1** **NR3C1** **SMAD4** MAP2K6 SMAD3 TGFBR2	**DUSP1** **IL8** **NFKBIA** **SMAD4** **TNF** IKBKB MAP2K6 SMAD3	Monocyte and its Surface Molecules	**ICAM1** **ICAM2** **CD44** **LTBR** **SELE** **CD83** **HLA-DOA** ITGAL PECAM1	
Role of Mitochondria in Apoptotic Signaling	**BCL2L1** **BID** **BIRC3** **CASP7** **ENDOG** CASP9 PDCD8		NF-kB Signaling Pathway	**IRAK1** **NFKB1** **TNFAIP3** **TRAF6** FADD	**IL1A** **MAP3K1** **NFKBIA** **TNF** **TNFAIP3** FADD	T Cytotoxic Cell Surface Molecules	**CD8A** **ICAM1** **LTBR** CD2 ITGAL PTPRC	
SODD/TNFR1 Signaling Pathway	**BIRC3** **TRAF2** FADD		Signal transduction through IL1R	**IL1B** **MAP2K3** **NFKB1** **TRAF6** IL1RAP MAP2K6	**IL1A** **MAP3K1** **NFKBIA** **TNF** IL1RAP MAP2K6	T Helper Cell Surface Molecules	**ICAM1** **LTBR** CD2 CD4 ITGAL PTPRC	
Stress Induction of HSP Regulation	CASP9 MAPKAPK3	**IL1A** **TNF** CASP9 CYC1	TNFR1 Signaling Pathway	**PAK2** **TRAF2** CASP2 FADD PARP1		The 4-1BB-dependent immune response	**IFNG** **NFKB1** **TNFSF9** **TRAF2**	
TNF/Stress Related Signaling	**MAP2K3** **NFKB1** **TANK** **TRAF2** CASP2 MAP2K6		TNFR2 Signaling Pathway	**DUSP1** **NFKB1** **TANK** **TNFAIP3** **TRAF2**	**DUSP1** **MAP3K1** **NFKBIA** **TANK** **TNFAIP3** **TNFRSF1B**	Toll-Like Receptor Pathway	**IRAK1** **MAP2K3** **MAP3K7IP2** **NFKB1** **TRAF6** MAP2K	
			
**Pathways**	**HIV-VLP vs PBS**	**HIV-VLP vs LPS**	**Pathways**	**HIV-VLP vs PBS**	**HIV-VLP vs LPS**			
			
**Cytokine Network**			**Phagocytosis**					
Cells and Molecules involved in local acute inflammatory response	**ICAM1** **IL8** **LTBR** CERKL ITGAL		Instracellular protein transport	APG9L1 RERE RNF4				
Cytokine Network	**IFNG** **IL18** **IL8**		**Cytoskeleton**					
Cytokines and Inflammatory Response	**CSF1** **CSF2** **IFNG** **IL7** **IL8** **IL15** **IL15RA** **IL23** **PTGS2** CD4		Cellular motiliyshape	**ARHGEF2** **ARPC2** **C14orf43** **FLJ39378** **MARCKS** **PDE4B** **PDE5A** **PLEK** **PPP1R15A** **PPP1R15B** **SLC11A2** **SLC1A3** **SLC20A1** **SLC43A3**				
IL 17 Signaling Pathway	**CD8A** **IL8** **CD58** CD2 CD4							
Th1/Th2 Differentiation	**IL18** **IL18R**							

The MDDCs differentiation process induced by HIV-VLPs, in fact, results in the reduced transcriptional levels of genes associated with phagocytosis and pathogen recognition. In parallel, an activation of genes associated with antigen presentation functions is observed. A set of cytoskeletal genes that may potentially mediate shape change and migratory behavior of activated DCs is also observed. The increase in the expression of immune cytokines, chemokines, and receptors contribute to the recruitment of monocytes, DCs, and macrophages to the site of infection. Moreover, they modulate both innate and adaptive immune response, including the polarization of Th cells, and the down-regulation of the inflammatory response, which may significantly interfere with the immune response. The induction of signaling genes and transcription factors may be involved in preparing the DC to be receptive to regulatory signals in the lymphatics and lymph nodes.

Finally, genes involved in the cell replication and apoptosis are modulated in their transcriptional levels, in agreement with the observation that mature DC undergo growth arrest, terminal differentiation and die by apoptosis 8–9 days after activation *in vivo*.

It is not unexpected that many pathways share same genes with intra-cellular functions involved in several biological outcomes. The majority (60%) of the pathways specifically modulated in the MDDCs by the HIV-VLPs treatment result from the comparison with the PBS treatment; the remaining 40%, instead, derives from the comparison with both PBS and LPS treatments.

### Functional implementation of activated genes in MDDCs in response to HIV-VLPs

The effects of the HIV-VLP treatment on MDDCs, compared to the PBS and LPS treatments, has been analyzed also considering the individual genes showing increased transcriptional levels. The presumed biological implication of selected genes involved in the specific functions of Dendritic Cells as professional APCs, has been evaluated in more detail according to their function (Table II).

**Table 2:** Functional categories of genes differentially upregulated in HIV-VLPs-induced MDDCs. (+) 1.5-fold upregulation; (++) 2–5-fold upregulation; (+++) >5-fold upregulation.

Pathway	HIV-VLP vs PBS	HIV-VLP vs LPS	Pathway	HIV-VLP vs PBS	HIV-VLP vs LPS
**Cell cycle, death**			**Chemokine/Immunology**		
TNF	++	+	*Innate response*		
BIRC3	++		IL8	+++	++
CASP7	+		CXCL1	++	++
BID	++		IL1a	++	++
PAK2	++		IL1b	++	
TRAF2	++		IL6	++	
BCL2L1	+		CCL4	++	
IGF1R	++		IFNG		+
TNFAIP3	++		PTGER4		
TANK	++		*Adaptive response*		
PSEN1	+		EBI2	++	
TNFRSF1B	+++	+	ICAM1	++	
**Transcription**			B2M	+	
NFKB1	+		*Receptors*		
NFKBIA		++	IL15RA	++	
IRAK1	+		IL18R1	++	
MAP3K1		+	IL23A	++	
JUNB	+		IL2RB	++	
**Signaling**			FCGR2A	++	
MAP2K7IP2	+		IL7R	++	
DUSP1	++	+	CD83	++	
MAP2K3	+		*Growth Factors*		
PAK2	++		CSF1	++	
RB1		+	CSF2	+	
FOXO3A	+		*Migration and Homeostasis*		
FYN		+	CCR7	+	
GNAQ	+		FPR1	++	
MEF2A	+		SELE	++	
RAPGEF2		+	CD44	++	
			LTBR	+	
			*T-cell activation/polarization*		
			CD40	++	
			TNFSF9		+
			IL18	+	

STAT2 is activated upon the binding of type-I IFN, the major component of the innate immune system, to its receptor and participates to the formation of interferon-stimulated gene factor 3 (ISGF3) complex, composed of STAT1, STAT2, and IFN regulatory factor 9, which promotes serial synthesis of selected proteins that inhibit viral replication [30,31].

Macrophage and Granulocyte-Macrophage Colony Stimulating Factor, CSF1 and CSF2, represent the main factors involved in proliferation and differentiation of myeloid lineage progenitor cells [32-35]. They may contribute to an increased production and recruitment of monocytes, DCs, and macrophages to the site of infection.

Formyl Peptide Receptor (FPR) is responsible for the DC chemoattraction to the Gram-negative bacteria Formyl peptide *N*-formyl-Met-Leu-Phe (fMLP) [35] and to additional pathogen-derived peptides, including HIV [36,37]. E-Selectin (SELE) and Chemokine (C-C motif) receptor 7 (CCR7) are selectively expressed on mature DCs, following encounter with pathogens. They favor the interaction with the endothelial cells as well as the migration from peripheral tissues to the T cell areas of secondary lymphoid organs, where they produce regulatory cytokines and prime naïve T lymphocytes [38,39]. Lymphotoxin-beta receptor (LTBR) plays a central role in the DC homeostasis in lymphoid tissues (spleen and LN), promoting and sustaining local DC replication [40]. Furthermore, a set of cytokine receptors that share a common γ chain (IL-2R, IL-7R, IL-15R, and IL-4R) were also induced and their expression may allow DCs to respond to lymphocyte-derived interleukins within the lymph node.

Beta-2-microglobulin (B2M) is one of the components of the multimolecular peptide-loading MHC class-I complex within the endoplasmic reticulum (ER) of DCs [41], representing the essential process for antigen presentation to cytotoxic T lymphocytes.

CD40 and TNFSF9 may act as co-stimulatory molecules which, interacting with the corresponding T cell expressed ligands, have been shown to play a relevant role in the activation of T cells. In particular, the CD40:CD40L interaction activates the CD4+ cells to promote the sensitization of CD8+ cells as well as to induce memory CD8+ T cell proliferation [42,43]. ICAM-1 is required on the surface of exosomes produced by mature DCs to prime naïve T cells and trigger effector T-cell response [44].

IL-18 has been shown to have a Th1/Th2 polarization effect. In particular, in the presence of IL-12, it enhances the IL-12-driven Th1 immune response; in the absence of IL-12, instead, IL-18 can stimulate Th2 cytokine production as well as allergic inflammation [45,46].

Collectively, these changes of gene expression in response to HIV-VLPs reflect a significant cellular and immunological reprogramming of the DC.

## Conclusion

The results here described confirm that baculovirus-produced HIV-VLPs induce a maturation pattern in MDDCs consistent with the one triggered by known activators, such us LPS, TNFα or cytokine cocktails. The genomic transcriptional profile induced by HIV-VLPs in MDDCs shows the activation of unique genes and cellular pathways compared to both PBS and LPS induction, reflecting a distinctive cellular and immunological reprogramming.

An unsupervised Eisen's clustering analysis confirm the specificity of the observation, given that the MDDCs samples derived from the HIV-VLP treatment cluster together. The only sample treated with HIV-VLPs which forms a different cluster on the basis of the 3,119 filtered genes, aggregates to the HIV-VLP samples when the differentially expressed genes are considered. This confirms that the pattern of genes specifically modulated by the HIV-VLP treatment is consistent all across the analyzed samples. Moreover, the genes involved in the transcriptional profile cluster according preferentially to the donor more than to the length of stimulation (4 or 8 hr), indicating that the induced modulation of transcriptional activity is effective after as early as 4 hours and is persistent for the length of the observation. Furthermore, the observed different donor susceptibility to HIV-VLP-treatment may suggest the possibility to select specific gene patterns useful for the identification of "responsive" vaccinees. This would be extremely helpful in understanding the eventual failure in individuals enrolled in the clinical trials.

Among the pathways and specific genes activated in MDDCs treated with HIV-VLPs, those directly involved in the biological functions as antigen presenting cells (APCs) have been analyzed in detail. The full functional maturation and activation of MDDCs by HIV-VLPs has been confirmed and, in particular, the activation of genes involved in cellular control (proliferation, differentiation, migration and homeostasis) as well as in functional activity (antigen presentation, T cell activation and Th polarization) has been observed.

The ability of the HIV-VLP, as exogenous antigen, to induce a CD8+ cytotoxic T lymphocyte (CTL) response, as previously demonstrated *in vivo*, is supported by the activation of genes (such as beta 2 microglobulin) involved in the antigen presentation within the context of major histocompatibility complex (MHC) class I molecules as well as in the Th1 polarization (IFN gamma and IL-18).

The presentation of an exogenous antigen in the context of major histocompatibility complex (MHC) class I molecules, to induce a CD8+ cytotoxic T lymphocyte (CTL) response, is referred to as "cross-presentation", in alternative to the "direct" or "classic" presentation route for endogenously synthesized proteins. The cross-presentation is a strategy employed only by DCs, among all the antigen presenting cells, to ensure an anti-virus CTL response also for those viruses which do not infect DCs [47]. The CD8+ DC subset seems to be primarily involved in this strategy [48,49], but the molecular mechanisms underlying the cross-presentation are not yet fully elucidated [50-52].

Microarray approach allows quantitative and simultaneous analysis of gene expression of a large amount of genes and the systematic studies of expression patterns are extremely useful for identify molecular events and key pathways involved in cellular functions induced by specific stimuli. In this study, informative data on the global pattern of gene expression underlying the activation of MDDCs by HIV-VLPs at the early stages of the immune response have been obtained. They may be extremely helpful for the identification of exclusive activation markers to trace the biological effects of modifications/optimizations of the HIV-VLP vaccination strategy.

**Figure 6:**
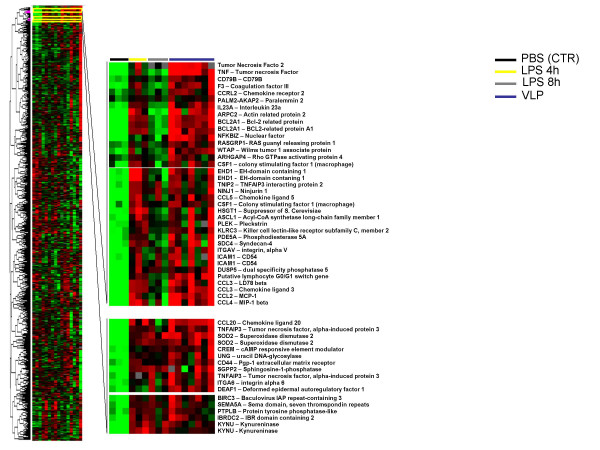
**Supervised hierarchical clustering of genes upregulated by HIV-VLPs and LPS in MDDCs**. 3,119 genes included in this analysis were filtered upon the criteria of showing less than 20% missing data and a minimum of 3-fold modulation in expression. The expanded section shows nodes including genes upregulated by HIV-VLPs and LPS-4 h (top) or by HIV-VLPs and LPS (bottom). Individual genes are indicated on the right.

**Figure 7:**
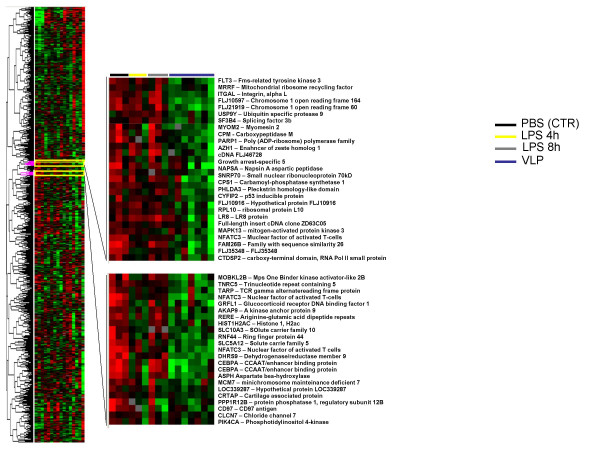
**Supervised hierarchical clustering of genes downregulated by HIV-VLPs and LPS in MDDCs**. 3,119 genes included in this analysis were filtered upon the criteria of showing less than 20% missing data and a minimum of 3-fold modulation in expression. The expanded section shows nodes including genes downregulated by HIV-VLPs only or by HIV-VLPs and LPS-4 h are shown (top and bottom, respectively). Individual genes are indicated on the right.
